# Supplementary material for: Platinum-bearing chromite layers are caused by pressure reduction during magma ascent
Source: Nat Commun. 2018 Jan 31;9:462. doi: 10.1038/s41467-017-02773-w (PMC5792441; doi:10.1038/s41467-017-02773-w)
Supplement: Supplementary file 2 — Description of Additional Supplementary Files [file 41467_2017_2773_MOESM2_ESM.pdf]

Description of Additional Supplementary Files

File Name: Supplementary Data 1
Description: AlphaMELTS isobaric runs for a basaltic liquid in Table 1 at a pressure range from 1 to 10 kbar.

File Name: Supplementary Data 2
Description: Summary of alphaMELTS isobaric runs for a basaltic liquid in Table 1 at a pressure range from 1 to 10 kbar.
